# Challenges and opportunities in the regulatory landscape of genetically modified crops in the European Union

**DOI:** 10.3389/fbioe.2025.1709118

**Published:** 2025-10-29

**Authors:** Monica Garcia-Alonso, Esteban Alcalde, Ine Criel, Concepción Novillo, Petra Kostolaniova, Nancy Podevin

**Affiliations:** ^1^ Estel Consult, Bracknell, United Kingdom; ^2^ Syngenta, Brussels, Belgium; ^3^ BASF, Ghent, Belgium; ^4^ Bayer, Madrid, Spain; ^5^ CropLife Europe, Brussels, Belgium; ^6^ Corteva Agriscience, Brussels, Belgium

**Keywords:** Genetically modified crops, regulatory framework, risk assessment, precautionary principle, proportionality, right to good administration

## Abstract

Genetically modified (GM) crops, introduced in the mid-1990s, have undergone extensive scientific scrutiny over the past three decades. While initial regulatory frameworks were stringent due to the nascent nature of the technology and uncertainty regarding their safety, subsequent comprehensive research and independent risk assessments have consistently affirmed their safety for human, animal, and environmental health, leading to widespread global adoption. GM crops have demonstrably enhanced agricultural productivity, mitigated environmental impacts associated with conventional farming, and contribute to the United Nations Sustainable Development Goals. Consequently, many nations have refined their regulatory approaches based on accumulated scientific evidence. However, the European Union (EU) presents a contrasting scenario. Despite significant investments in agricultural biotechnology research and a commitment to the Sustainable Development Goals, the EU’s regulatory landscape for GM crops has become increasingly complex, effectively limiting European farmers’ access to these technologies. The EU heavily relies on imports of GM-containing protein-rich crops, while its farmers cannot benefit from their cultivation. The current pre-market assessment for GM crop import and food and feed use authorization is characterized by its lengthy, costly, and unpredictable nature. This paper discusses some of the issues faced by developers when trying to obtain import approvals for GM crops in the EU and the impact that the current regulatory framework is having on innovation. The authors propose a series of practical and feasible adjustments that could be implemented during the EU’s risk assessment process to unlock the benefits of biotechnology for European agriculture without compromising safety standards.

## Introduction

Agricultural biotechnology was a nascent technology in the early 1990s, with the first genetically modified (GM) crops entering the food market in 1996. At the time, there was uncertainty regarding their potential safety impacts on humans, animals and the environment, and this led to many countries setting stringent regulatory frameworks, many of which were initially based on the precautionary principle. Now nearly three decades later, GM crops have been planted in 27 countries over 209.8 million hectares (Agroinvestor, 2025). Extensive research has shown that GM crops have increased global production of food, feed and fibre and have assisted farmers in reducing the environmental impact associated with their crop protection practices and carbon emissions (Brookes, 2022; Brookes and Barfoot, 2020). Hundreds of independent risk assessments conducted by regulatory authorities worldwide have consistently concluded that GM crops are as safe as their conventional counterparts for human and animal consumption and for the environment (Goodman, 2024; Smyth et al., 2021). Consequently, many countries have streamlined their regulatory framework for GM crops, considering scientific evidence and their own regulatory experience. Unfortunately, this has not been the case in the European Union (EU), where GM crop regulations kept increasing in complexity over time (Garcia-Alonso et al., 2022).

The benefits delivered from the adoption of GM crops align with the United Nations Sustainable Development Goals (SDGs) (Lee et al., 2016). The EU has fully committed to achieving these SDGs and has invested in scientific, technological, and industrial excellence with the aim of fostering innovation to fulfil these goals (European Commission, 2024a; European Commission Directorate-General for Research and Innovation, 2010). However, despite substantial investment in research and development, most European farmers do not have access to GM crops. This is primarily due to the regulatory oversight for these products posing major challenges for developers (Masip et al., 2013; Raybould and Poppy, 2012). In contrast, the EU depends heavily on the importation of protein-rich crops from the Americas. Given the high adoption rate of GM crops outside the EU, most of these imported commodities inevitably contain GM crops. Consequently, while food and feed derived from GM crops is consumed in the EU, EU farmers cannot benefit from this technology and remain reliant on traditional crop protection methods or agricultural practices with higher environmental impacts (Masip et al., 2013).

Under EU legislation, food and feed products containing or produced from GM crops undergo a pre-market assessment for import authorisation. This process is lengthy, costly and unpredictable (Garcia-Alonso et al., 2022), an issue that the European Commission (EC) has identified as a key challenge to be addressed for fully utilizing biotechnology innovation to enhance EU industrial competitiveness and sustainability (European Commission, 2022; European Commission, 2024a).

While a legal review of the current GMO regulations for GM plants in the EU is not anticipated in the short-term, practical adjustments to the current system, particularly to the risk assessment process, are feasible without compromising safety.

This paper offers an industry perspective of the regulatory hurdles faced by developers of GM crops in the EU, explores the global implications of these challenges, and discusses opportunities for adaptations within the existing legal framework.

## EU regulatory landscape for GM crops

The introduction of GM crops in the late 1990s coincided with unrelated food crises in Europe, such as bovine spongiform encephalopathy (BSE), contributing to a decline in public trust over scientific advice and governance that resulted in a negative perception of EU food safety oversight. This was particularly punitive for GM crops, a nascent technology at the time, where stringent regulations based on the precautionary principle were introduced (Anyshchenko and Yarnold, 2021; Garcia-Alonso et al., 2022). Over time, EU GM crop regulations increased in complexity culminating with the introduction of the Commission Implementing Regulation (EU) No 503/2013 (European Commission, 2013), hereafter referred to as the Implementing Regulation. This regulation, based on the European Food Safety Authority (EFSA) guidance developed in 2011 (EFSA, 2011b), made data requirements for GM crop risk assessment legally binding and introduced additional requirements, such as mandatory animal testing (Garcia-Alonso et al., 2022).

The European Union’s primary legislation establishes fundamental principles that apply to all sectors regulated under its jurisdiction. These principles encompass the precautionary principle (European Commission, 2012b), the principle of proportionality (European Commission, 2012b), and the right to good administration (European Commission, 2012a). EU legislation also underscores core values such as animal welfare, the reduction of animal testing, and the efficiency and sustainability of risk assessments, and is currently encouraging activities that support regulatory simplification aimed at boosting innovation (European Commission, 2010; European Commission, 2022; European Commission, 2024a; European Commission, 2024b).

The precautionary principle, as defined in Article 191 of the Treaty on the Functioning of the European Union (European Commission, 2012b) allows for preventative action in the face of potential risk uncertainty to prevent harm to the public or the environment. It is relevant when scientific evidence is inconclusive and there are concerns about potential adverse effects, but not when sufficient scientific evidence exists, and the risk assessment body has not identified unacceptable levels of uncertainty.

The principle of proportionality, as laid down in Article 5(4) of the Treaty on European Union (European Commission, 2012b) and embedded in the General Food Law (European Commission, 2002), mandates that regulatory burdens should be proportional to the risk that they aim to mitigate (Van der Meulen, 2010). Article 5 of the General Food Law (European Commission, 2002) also specifies that regulatory burdens must be justified by the level of risk.

The right to good administration, found in the EU Charter of Fundamental Rights (European Commission, 2012a), highlights the need for effective and sustainable risk assessments, and the Transparency Regulation (EU) 2019/1381 (European Commission, 2019) further emphasizes the importance of sustainability and efficiency in risk assessment.

The EU Directive 2010/63/EU, also known as the 3R legislation (European Commission, 2010) grounded in Article 13 on the treaties on the functioning of the EU, focuses on the reduction of the number of animals used for experimental purposes and their welfare. It promotes the Replacement, Reduction, and Refinement of animal use, ensuring that animal testing occurs only when absolutely necessary and is justifiable from a scientific or educational point.

It is the view of the authors that these principles are not applied coherently in the regulation of GM crops, especially considering the large body of scientific research supporting the inference that GM crops are as safe as their conventional counterparts (European Commission, 2010; Devos et al., 2016; Goodman, 2024; Smyth et al., 2021). While invoking the precautionary principle may have been understandable in the early years of GM crops, it is no longer sensible to apply it after three decades of safe use. Nevertheless, the EU has not updated its approach and has instead continued adding more stringent prescription (Garcia-Alonso et al., 2022). The European Commission has recently acknowledged the need for balanced regulation (European Commission, 2024b) to promote the competitiveness of biotechnology and biomanufacturing industries, and to streamline EU legislation to reduce fragmentation, simplify regulatory processes, and accelerate time to market for biotechnology innovations (European Commission, 2024a; European Commission, 2010). This indicates a willingness within the EU to support EU scientific developments and to establish a balanced and proportionate regulatory framework for biotechnology products. Legislative changes in the EU system can be lengthy and require arduous negotiations. However, adaptations within the current legal framework to improve the regulatory process for GM crops are possible and would align with current EU goals (European Commission, 2024a). The following sections describe some of the regulatory hurdles faced by developers trying to obtain GM crop import approvals in the EU and provide examples of potential adaptations that could make the system more efficient.

## Regulatory hurdles under the current EU system

In the EU, developers face significant challenges in obtaining approvals for the import and food and feed use of GM crops under the Implementing Regulation (European Commission, 2013). While this regulation generally aligns with the Codex Alimentarius guidance (Codex Alimentarius, 2009-which is followed by many other countries and international organizations) it includes additional data requirements and methodology prescriptions unique to the EU.

Applications for the authorisation of GM crop imports must be submitted to the European Food Safety Authority (EFSA) and must adhere to EFSA’s administrative guidance (EFSA, 2021). EFSA performs a completeness check, ensuring that data requirements specified in the Implementing Regulation (European Commission, 2013) are met and that data have been generated according to the latest EFSA guidance documents (EFSA, 2025), which provide detailed instructions for study design, data generation and statistical analyses. If data are missing or deviate from EFSA’s methods, applications are put on hold until all requirements are fulfilled. For some developers, this process takes several years.

The EC and EU Member States rely on EFSA to review these applications and conduct the risk assessments. However, EFSA’s current approach limits flexibility for case-by-case assessments or risk assessments proportionate to the risk posed by a particular GM crop. This is mainly due to EFSA’s interpretation of the Implementing Regulation (European Commission, 2013) data requirements and of the role that EFSA has been given by the EC in ensuring its compliance. Notably, the Implementing Regulation (European Commission, 2013) includes an article, Article 5(2), that allows developers to request exemptions (derogations) from data submission requirements if there is a valid justification. Justifications should be based on: 1) the nature of the genetic modification or product, and 2) the scientific necessity of the information for risk assessment or technical impossibility of obtaining the data required. This is referred to as the “derogation clause”, which offers a viable mechanism for case-by-case adaptation of the risk assessments based on the product characteristics.

However, in practice, EFSA rarely accepts derogations. Since the Implementing Regulation came into force, EFSA has been adding more prescriptive guidance and technical papers aimed at harmonising all GM applications. The review of GM crop applications at EFSA is conducted by an independent panel of experts (GMO panel) and overseen by EFSA staff. The GMO panel is renewed every 4 years, with a time limit on participation. Over time, developers have observed that when new members join EFSA’s GMO Panel, changes in interpretation and implementation may occur. These adjustments are typically connected to prospective research interests and can lead to the development of new guidance or requests for supplementary information beyond the requirements set forth by CODEX and Implementing Regulation. For developers, it is a fundamental issue as the generation of regulatory data takes years and any changes in guidance introduced while the data package is being built may result in delays, making the system unpredictable due to constantly changing goalposts.

The continuous proliferation of EFSA guidance adding more data requirements and more prescription is also driven by the aim to harmonize all applications, irrespective of their risk profile, specificity, availability of scientific data, and results of previous risk assessments. As a result, the review of applications has become a one-size-fits-all checkbox exercise. This approach is inconsistent with the case-by-case approach, EU primary legislation principles described in this paper and the EC’s aspiration to streamline regulation.

Notably, this regulatory system has led to market concentration, limiting the number of developers able to navigate it successfully. Those who succeed, tend to focus on products with the potential to recoup the high regulatory costs associated with EU applications. This poses a significant challenge to market for innovative niche products or products developed by Small and Medium-Sized Enterprises (SMEs) and public institutions.

## Specific examples of regulatory obstacles

### Stacked GM events obtained by conventional crossing

One area that really singles out the EU regulatory approach to GM crops is the regulation of products resulting from conventional crossing of two or more GM events, commonly referred to as “stacked events” or “stacks”. Most jurisdictions worldwide consider conventional breeding as a safe practice, and once the safety of a single transformation event (single GM event) is established by regulatory agencies, subsequent conventional crosses, whether with GM or non-GM varieties are generally considered safe and do not require further regulatory oversight. In some countries additional assessment may be required if the crosses involve two or more single GM events with a reasoned scientific hypothesis for trait interactions that could potentially cause adverse effects (Goodwin et al., 2021). However, in the EU, the conventional crossing of two or more single GM events is regulated as a new GM crop.

The Implementing Regulation mandates that the risk assessment for GM stacks includes a risk assessment of each single GM event. It also requires an evaluation of the stability of the transformation events in the stacks, a comparison of the expression levels of the newly expressed proteins in the stack and each single event and an assessment of potential synergistic or antagonistic effects resulting from the combination of the single events. The Implementing Regulation also states that compositional analyses and agronomic and phenotypic comparisons with the stack may have to be conducted.

In the EU EFSA does not start the risk assessment of stack applications until the risk assessment of each of the single events has been completed (Garcia-Alonso et al., 2022). This approach has a significant impact on the review timelines. While cultivation approval for a single GM event takes on average one or 2 years in most countries, import and food and feed applications in the EU average 4 years from submission to approval (Garcia-Alonso et al., 2022). The review of stack applications in the EU takes a similar amount of time as single GM events. In addition, severe delays can occur if EFSA issues new or updated guidelines between the review of a single event and its inclusion in a stack application, as these changes are often applied retroactively and studies may have to be repeated (Roper et al., 2022).

The accumulated global regulatory experience with GM stack assessments and multiple independent studies and publications indicate that stacking GM events by conventional breeding does not increase the risk of the single events (Goodwin et al., 2021). This approach has been a long-standing policy at the United States Department of Agriculture (USDA), the United States Food and Drug Administration (FDA), Health Canada, China and the Food Standards Australia and New Zealand (FSANZ), where no additional safety assessment is conducted for stacks of previously approved single GM events. In view of the regulatory experience gained with safety assessments for stacks, some countries like Japan have updated and streamlined their regulations implementing an approach whereby familiarity with the events and types of combinations determine whether further regulatory oversight is needed (Iizuka, 2020; Kasai and Ohsawa, 2021).

Argentina has also undertaken a comprehensive review of its agricultural biotechnology regulatory policies, drawing on its extensive experience and continuous engagement with advances in scientific knowledge. This revised framework has led to more streamlined and predictable oversight procedures, greater accessibility for small and medium-sized enterprises and academic institutions seeking approvals, and an expanded diversity of approved events and crop species (Lewi et al., 2025).

Streamlining the risk assessment for stacked events obtained by conventional crossing would align with the current EU principles and objectives mentioned above (European Commission, 2010; European Commission, 2022; European Commission, 2024a; European Commission, 2024b). These products are grown in cultivating countries, as they are considered valuable tools for sustainable agricultural production. Delays in obtaining approvals in the EU lead to trade issues and hinder innovation.

Given EFSA’s extensive experience with stacked events reviews and the well-documented safety of conventional crossings, streamlined procedures should be considered for stacked events obtained by conventional crossings for which the risk of the parental events was assessed by EFSA, without compromising safety. Following the Implementing Regulation, stack submissions could comprise cross-references to relevant data previously submitted for each single event, data on stability and expression, and an assessment of potential interactions between events that could impact food and feed safety. These initial assessments would determine whether additional data on the stack (e.g., composition or agro-phenotypic comparisons) are warranted. Derogations for any study that would not be necessary from a scientific point of view should be accepted. This approach would be more in line with the EU principles of proportionality and good administration and the EC’s aim to simplify regulatory procedures that foster innovation in biotechnology. It would also align with the approach used in other countries, such as Japan, Brazil, and Argentina, where data on stacks is not required unless interactions that could lead to adverse effects have been identified. It is imperative to note that to this date, interactions between traits present in different GM single events in a stack have not been identified or reported.

### Balancing animal welfare and scientific risk assessment needs

The Implementing Regulation mandates animal testing for all new single GM events (European Commission, 2013). This EC requirement was aimed at facilitating consensus among Member States (Garcia-Alonso et al., 2022), and therefore was driven by political motives rather than scientific reason. This requirement has been a subject of ongoing debate. EC-funded research programmes concluded that 90-day feeding studies for GM crops do not add valuable data to risk assessments in the absence of a clear testing hypothesis (European Commission, 2018). EFSA has supported these conclusions on multiple occasions (Devos et al., 2016), but also interpreted the EC decision as a *de-facto* regulatory requirement for all GM single events (EFSA, 2011b). In addition, while international guidelines for performing 90-day rat toxicity studies have been in place since 1981 under the Organisation for Economic Co-operation and Development (OECD) umbrella (OECD, 1998), EFSA opted to publish their own guidelines in 2014 (EFSA, 2014). This rigidity has led to situations where EFSA has rejected studies performed under OECD guidelines and requested that the studies be performed using EFSA methodology.

An example is provided in Roper et al. (2022), where a 90-day rat feeding study had been performed to support the application for food and feed approval of GM maize line TC1507 in 2002. The study was compliant with OECD guidelines (OECD, 1998) and EU regulatory requirements at the time. The study was evaluated by EFSA and considered acceptable, consequently, TC1507 was approved in the EU in 2004. The same study was later used to support stack applications containing TC1507 and accepted. However, when more applications for approval of stacks containing TC1507 were submitted in 2015, EFSA requested a new 90-day rat feeding study, arguing that the existing study did not comply with the latest EFSA guidance (EFSA, 2014) and therefore could not support a risk conclusion. A derogation based on the fact that a valid study already existed was not accepted. This represents a clear deviation from EU principles, especially from the 3R legislation (Replacement, Reduction and Refinement of animal use) (European Commission, 2010).

Considering the reaffirmed EU commitment to phase out the use of animals for testing purposes, an alignment between the EC, EU Member States, and EFSA is imperative. A practical short-term solution would be for the EC to enable EFSA to conduct science-based, case-by-case risk assessments, where studies that follow international guidelines and are scientifically sound, are accepted, even when the guidelines deviate from EFSA guidance. Also, there is abundant scientific evidence that confirms that 90-day rat feeding studies do not add value to risk assessments, unless there is a clear risk hypothesis (Devos et al., 2016; European Commission, 2018). Article 5 in the Implementing Regulation 503/2013 could be used to accept derogations.

### Comparative assessment of crop composition in the EU

In the EU, deviations from primary legislation principles are also evident in the comparative assessment conducted to analyse and compare crop composition. These studies are recommended by the Codex Alimentarius guidelines (Codex Alimentarius, 2009) as part of the hazard identification process for food safety assessments. The aim is to compare the composition of key nutritional parameters in a conventional crop with the GM version of the crop to determine whether the genetic modification has led to unintended changes in composition. Samples for analyses are collected from regulatory field trials and analysed in certified laboratories.

While most countries request these studies only for GM single events, EFSA also requests these studies for stacks. In addition, there are remarkable differences in how these studies are conducted in the EU and in the rest of the world. As previously discussed, the Implementing Regulation (European Commission, 2013) was based on existing EFSA guidance, providing detailed instructions on the design and statistical analyses to follow for compositional analyses (EFSA, 2010; 2011a), which has since been complemented with further guidance (EFSA, 2015; 2018). For instance, the studies must be conducted at a minimum of eight sites, with four replicates per site (8x4) and must include a comparison of the GM plant with its conventional counterpart and with a minimum of three non-genetically modified reference varieties of the crop per site (with a minimum of six over the whole study). When genetically modified plants possess herbicide-tolerant traits, composition data are required for both treated and untreated GM entries. This translates to a minimum of five entries, replicated four times on at least eight sites, resulting in 160 entries for which approximately 80 analytes (in the case of maize) are measured, leading to a minimum of 12,800 analyses. Should weather conditions or other circumstances compromise sample collection from any plot, EFSA deems the data set incomplete and requests additional data, leading to long delays and increased costs. Consequently, applicants routinely add extra sites, replicates, and/or number of commercial references. However, even when the number of entries for each line exceeds that derived from a perfect 8 by 4 design and the statistical power of the experiment is higher, EFSA does not accept data which does not meet the perfect 8 by 4 requirements.

In contrast, other countries make risk assessment conclusions based on expert judgement of studies that may include fewer sites and/or replicates, provided that the study design is scientifically sound (Brune et al., 2013) and do not impose the requirement of adding extra conventional varieties. A typical study may have two entries (GM and comparator), four replicates per entry at four sites (32 entries), resulting in 2,560 analyses, five times less than the minimum requirement in the EU. The results are then put into the context of natural variability (Codex Alimentarius, 2009) using the Crop Composition Database (AFSI, 2024), which contains compositional data on hundreds of varieties of many crops, to ascertain the biological relevance of any differences found. In contrast, EFSA imposes a complex statistical analysis that not only tests the hypothesis of no difference to the conventional counterpart, but also the equivalence of the GM crop with at least six reference varieties included in that study. This approach results in a variety of potential outcomes regarding differences and equivalences, with limited added value for the risk assessment. This begs the question of why EFSA cannot make a risk conclusion when a dataset is not produced exactly as prescribed when regulatory authorities around the world can reach a conclusion with a fraction of the data.

Furthermore, the EU is the only jurisdiction where such complex compositional analyses for stacked events are mandated. The costs and time associated with this endeavour can be borne only by large companies for products with a substantial market share. Smaller developers or developers of products that primarily focus on addressing challenges in niche markets are discouraged by this regulatory requirement, effectively hindering innovation.

Experience accumulated over 30 years supports the fact that genetic modifications introduced in commercial GM crops to date have not resulted in unexpected alterations of crop composition, while at the same time, differences can be clearly found between different conventional varieties of the same crop (Herman and Price, 2013).

This illustrates that EU’s mandatory data requirements conflict with the EU principles of proportionality and good administration and certainly do not align with regulatory simplification. It also shows that the acceptability of data by EFSA is contingent upon literal compliance with the Implementing Regulation and the prescribed EFSA methodology. There are, however, potential approaches that could be adopted by EFSA to improve the efficiency of the review process:

• For single GM events, existing compositional analyses conducted in the countries where these events were approved for cultivation could be accepted in the EU for import applications, even if the studies were not conducted in accordance with EFSA guidance. This approach would avoid duplication of data without compromising safety assessments. Aligning with the rest of the world would alleviate the burden associated with this intricate study design and result in data packages more representative of the level of risk posed by these products.
• For GM stacks where compositional analyses conducted with the single events have not revealed any biologically relevant changes in composition, and interactions among the single GM events that could lead to adverse effects relating to food or feed are unlikely, compositional analyses with the stack should not be mandatory. Extensive scientific evidence and a large number of compositional studies with stacks reviewed by EFSA confirm that conventional crossing of single events does not result in relevant compositional changes in stacks. In this case, the derogation clause could be used to avoid generating large data sets that are unlikely to provide additional information for the risk assessment.

These adaptations would be in line with the EU principles of proportionality and good administration and the EC’s aim to simplify regulatory procedures.

## Discussion and proposals for improvement

Scientific evidence and regulatory experience accumulated over the last three decades have shown that GM crops are as safe as their conventional counterparts. While polities that foster innovation in agricultural biotechnology have leveraged this experience and streamlined their regulatory oversight for these crops, the EU has continued to apply an overly precautionary approach that has led to regulations that deviate from key EU principles that are supposed to be applied across all regulated sectors. While the Implementing Regulation introduced in the EU in 2013 (European Commission, 2013) was intended to improve the approval process, its legally binding nature combined with the way in which it is currently implemented by EFSA has resulted in a cumbersome, costly, unpredictable and lengthy regulatory system, disproportionate to the risk posed by these products. More specifically, the current regulatory system does not build on the accumulated scientific evidence and regulatory experience gained over the last three decades and is not aligned with current EU objectives that aim at regulatory simplification to foster innovation in biotechnology.

While a legal review of the current legislation for GM plants in the EU is not anticipated in the short-term, practical adjustments can be made to modernise and streamline the regulatory process without compromising on safety. This is particularly important considering the pressing need to address global food supply challenges and the rapid pace of scientific discoveries, which are yielding new products that can significantly contribute to alleviate those challenges, benefiting both farmers and consumers.

A key tool at hand is Article 5 on derogations in the Implementing Regulation (European Commission, 2013). Its use could streamline risk assessments within the current regulatory framework, but this would require closer alignment between EU risk assessors (in EFSA) and risk managers (in the EC). Currently, EFSA appears to operate within the constraints of the mandatory and legally binding nature of the Implementing Regulation. As discussed in this paper, this approach has resulted in a trend where conducting science-based case-by-case risk assessments, proportionate to the risk posed by a particular product, is just not possible. Instead, EFSA has opted for a “harmonization” approach to ensure compliance, where it is expected that all GM applications for import and food and feed approval contain the same data, and that the data has been generated in the same prescribed manner. By enabling EFSA’s application of more modern approaches in risk assessment, building on scientific evidence and allowing the use of Article 5 on derogations, the EU could move closer to its aim of fostering innovation in this area. This would also result in assessments aligned with the EU principles used in other EU regulated products sectors and international best practices.

There are several ways in which the current regulatory system for GM crops could be improved within the existing legal framework:

• Streamlining the review for stacked products produced by conventional crossing.
  • Following a stepwise approach, where the data mandated by the Implementing Regulation for all stacks (genetic stability, expression and potential interactions) is examined and used to determine whether additional data is necessary.
  • Allowing the use of Article 5 derogations when there is scientific justification for not conducting additional studies.
• Aligning the risk assessment of GM crops with the 3R regulation that supports animal welfare and aims at minimising the use of animal testing.
  • Accepting existing animal studies conducted following international guidelines to avoid unnecessary duplication of studies.
  • Allowing the use of Article 5 on derogations when there is a scientific justification for not conducting such studies.
• Adopting a standardized approach for evaluating the reliability of study data across all regulated food areas, based on scientific principles such as the Klimisch score (Klimisch et al., 1997).
  • Aligning EFSA’s approach with other EU risk assessment bodies, ensuring that scientifically sound results are appropriately considered in risk assessments, even if they deviate from EFSA’s prescribed methodology.
• Ensuring a high level of competency in risk assessment methodologies in all personnel and panel experts involved in the review of GM crop applications. This would avoid questions and data requests driven by scientific curiosity rather than risk assessment best practices (“need to know” versus “nice to know” (Raybould et al., 2012)).

The authors acknowledge that EFSA is currently exploring ways to introduce regulatory simplification and are reviewing the extensive list of guidance documents with the aim to provide clearer procedures for developers. This effort is critical and should consider alignment with international best practices and approaches used by other EU risk assessment bodies. With some practical adjustments in the implementation of the current regulation for GM crops, the EU has the opportunity to move towards greater regulatory efficiency and unlock the current barriers to innovation in agricultural biotechnology, without compromising on consumer, animal and environmental safety.
